# MRI morphological evaluation of humeral head bone profile inside region of the biceps pulley reflection

**DOI:** 10.1007/s00256-022-04056-y

**Published:** 2022-04-22

**Authors:** Michele Fischetti, Luca Pio Stoppino, Miriana Rosaria Petrera, Alessia Francavilla, Roberta Vinci, Marcello Zappia, Luca Macarini

**Affiliations:** 1 Department of Medical and Surgical Sciences, University of Foggia, Foggia, Italy; 2 Department of Medicine and Health Sciences, University of Molise, Campobasso, Italy

**Keywords:** Humeral head anatomical variants, MRI shoulder, Tendon long head of biceps brachii

## Abstract

**Objective:**

Evaluating humeral head bone profile inside biceps reflection pulley area in order to identify possible anatomical variants and any causes predisposing to tendon’s instability of the long head of the biceps.

**Materials and methods:**

This retrospective study analyzed 326 patients, 183 males and 143 females (age 15–88 years; average 51.5 years), who underwent MRI examination between 2013 and 2019. Biceps pulley reflection area morphology of 192 right shoulders and 134 left shoulders was assessed analyzing 309 MRI and 17 MR arthrography (MRA) shoulder exams. We investigated age and gender and the frequency of morphological variants among the patient groups.

**Results:**

Four possible morphological variants were identified: 95 with convex shape; 127 with flat shape; 77 with spiculated shape; and 12 with mixed morphology. Fifteen humeral bone profiles were not classifiable.

**Conclusions:**

MRI was effective in defining humeral head anatomic variants inside the biceps pulley reflection area. The most frequent variants were flat or convex types.

## Introduction

The long head of the biceps tendon (LHBT) originates from the supraglenoid tubercle, and partly, from the glenoid labrum. Lesions affecting the LHBT are considered the most frequent causes of anterior pain and disability in the shoulder, with other less common causes including rotator cuff disease involving the subscapularis and supraspinatus tendons [1].

The LHBT passes across the “rotator interval” (i.e., intracapsular portion) before entering the bicipital groove (i.e., extracapsular portion) [2, 3]. In its course within the rotator interval, the LHBT is stabilized by the biceps pulley, a capsular-ligamentous complex formed by the coraco-humeral ligament (CHL) and superior gleno-humeral ligament (SGHL) [4].

The diagnosis and treatment of the pulley lesion, a potential cause of shoulder pain and dysfunction, represent a challenge for the radiologist and the orthopedic surgeon. The particular anatomic course of the LHBT explains its function, since as a sliding tendon, it pulls like a mechanical belt around the humeral head. Thus, it is stressed by traction, pressure, friction, and shearing forces. Apart from its close anatomic relationships with the rotator cuff, the sulcus of bicipital groove and acromion serves as additional potential sites of impingement, which may lead to secondary degenerative changes in the tendon.

To our knowledge, no study has previously evaluated the morphology of the humeral head at the region of the biceps pulley reflection (i.e., point where LHBT reflects from intra- to extra- articular portion at pulley level) which may reflect an additional potential cause of LHBT instability.

This study aimed to classify the morphological variants of biceps pulley reflection area observed during MRI shoulder examinations and to analyze the frequencies and demographic characteristics for each type of variant, in order to identify those predisposing to tendon’s instability of the long head of the biceps.

## Materials and methods

### Patients

We retrospectively reviewed 326 cases of MRI shoulder examinations that were performed in our university hospital between 2013 and 2019. A total of 183 male patients and 143 female patients aged between 15 and 88 years (average 51.5 years) were included and they were divided into three groups according to age: ≤ 25 years, 25–50, and ≥ 50 years. Primary patient complaints were shoulder pain with or without limitation of movement and shoulder dislocation. Exclusion criteria were marked signs of degenerative arthropathy (osteophytes, bone sclerosis, and subchondral cysts); bone fractures (T1 hypointense fracture line, bone marrow, and soft tissue edema); tumor lesions (lytic or sclerotic bone lesions) of the shoulder; and qualitatively limited examinations (i.e., patient movement, magnetic susceptibility artifacts). Approval from the Institutional Review Board was obtained and in keeping with the policies for a retrospective review, informed consent was not required.

### MRI protocol

All studies were obtained with a 1.5-T scanner (Achieva, Philips Healthcare, Best, the Netherlands) with a dedicated surface coil (Flex-M). Patients were positioned with their arms in a neutral position. The morphology of the humeral head bone profile inside biceps pulley reflection area of 192 right shoulders and 134 left shoulders was assessed by analyzing 309 non-contrast MRI and 17 MR arthrography (MRA) examinations.

For non-contrast MRI, axial gradient echo T2-weighted (T2w) sequence using fast field-echo (FFE) technique, oblique coronal proton density weighted (PDw), and PDw fat-saturated turbo spin-echo (TSE) images in the oblique coronal and oblique sagittal planes were obtained.

The MRA examinations were performed after intra-articular injection of approximately 15–20 ml of paramagnetic contrast (pre-filled 20-ml syringe of Gd-DOTA 2.5 mmol/l; Dotarem, Guerbet, France) via an anterior approach using a 22-gauge spinal needle. For MRA, axial 3D T1w gradient echo sequence with fat saturation (T1 High Resolution Isotropic Volume Excitation (THRIVE)), T1-weighted (T1w) TSE, and fat-saturated T1w TSE in the oblique coronal and oblique sagittal planes were obtained. MRA examinations were retrospectively selected and included in the study since the use of intra-articular paramagnetic contrast agent does not affect the imaging evaluation of humeral head morphology. Non-contrast MRI and MRA imaging parameters are summarized in Tables 1 and 2.

**Table 1:** Non-contrast MRI imaging parameters

**Non-contrast MRI**	Axial T2-FFE	Coronal PD-TSE	Sagittal and coronal PD-TSE-SPIR
Repetition time (ms)	863	1303	2218
Echo time (ms)	13.8	30	25
Echo train length	1	5	4
Flip Angle	35°	90°	90°
Acquisition matrix number	256 × 256	256 × 256	256 × 256
Slice thickness (mm)	3	3	3
Field of view (mm)	180	180	180
Number of averages	2	4	4

**Table 2:** MRA imaging parameters

**MR arthrography**	Axial 3D THRIVE	Sagittal and coronal T1-TSE	Sagittal and coronal PD-TSE-SPIR
Repetition time (ms)	9,8	533	2218
Echo time (ms)	4,9	20	25
Echo train length	30	5	4
Flip Angle	7°	90°	90°
Acquisition matrix number	512 × 512	256 × 256	256 × 256
Slice thickness (mm)	1.5	3	3
Field of view (mm)	180	180	180
Number of averages	2	6	4

### Image analysis

Images were independently evaluated by two experienced musculoskeletal radiologists (10 years for reader 1: LPS; 3 years for reader 2: MF).

MR images were analyzed on our picture archiving and communication system (CarestreamVue PACS—Carestream Health, Inc, Rochester, NY), using the sequences acquired on the axial plane (i.e., T2w-FFE and T1w 3D-THRIVE sequences), the most suitable for an objective and reproducible evaluation of the biceps pulley reflection area morphology. Cases which demonstrated humeral head position deviating from neutral were not felt to affect morphologic evaluation.

**Fig. 1:**
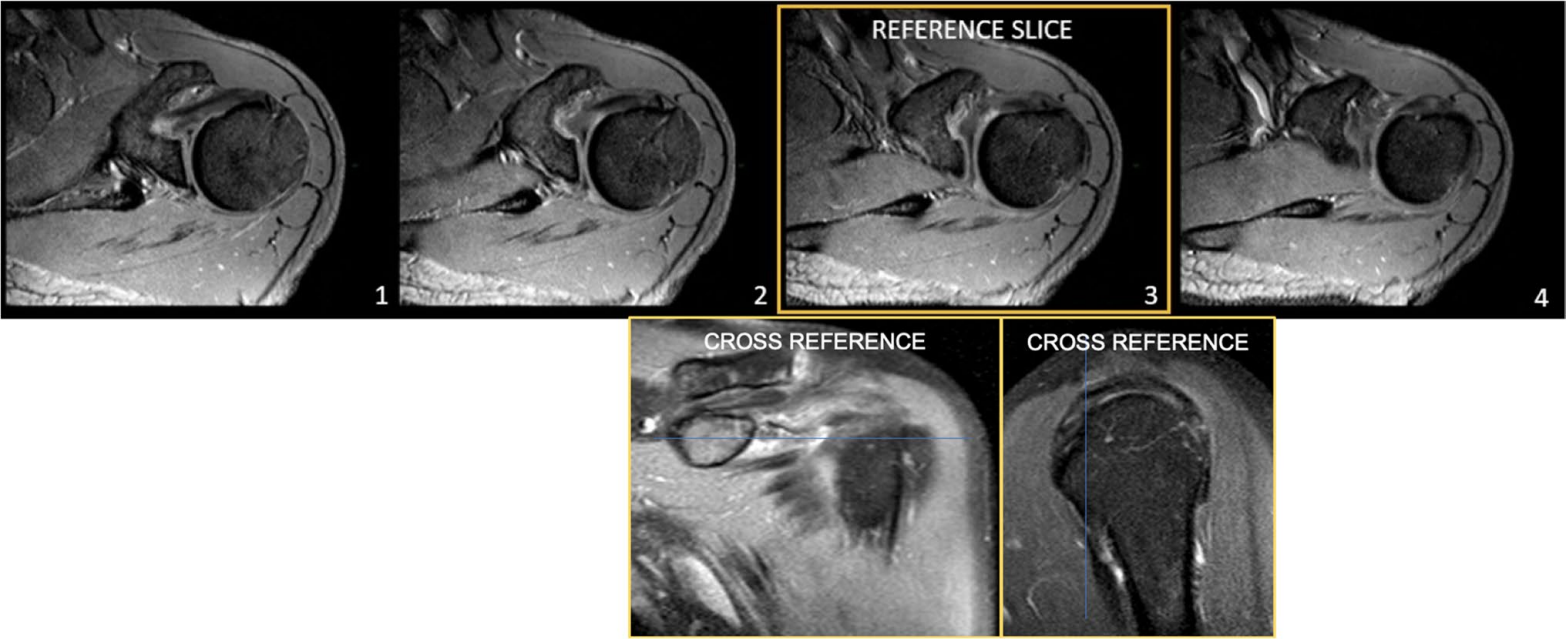
Axial T2w-FFE sequences showing the reference slice selected by drawing a virtual plane at the great tuberosity, just above the proximal end of the bicipital groove. On cross reference PDw fat-sat TSE images in the oblique coronal and oblique sagittal planes, the same area of interest is displayed

The reference slice (i.e., the slice used for morphological analysis) was selected by drawing a virtual plane at the great tuberosity, just above the proximal end of the bicipital groove. After a careful evaluation of the axial sequences, this seemed to be the one in which the point of passage from intra-to extra-articular portion of LHBT could be defined with greater precision (Fig. 1).

**Fig. 2:**
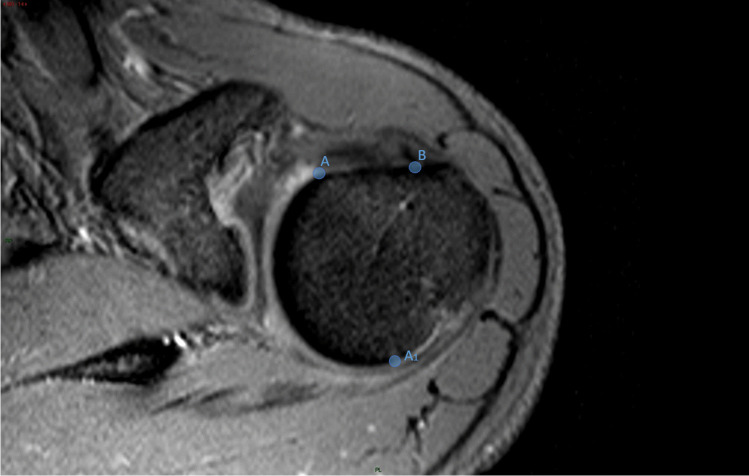
Axial T2w-FFE sequence showing the landmarks identified on the reference slice. A and A1: points beyond which the sphericity of the humeral head is outlined; B: point below which the bicipital groove starts

These landmarks were identified on the reference slice (Fig. 2):

- *A* and *A1*: points beyond which the sphericity of the humeral head is outlined.
- *B*: point below which the bicipital groove starts.

**Fig. 3:**
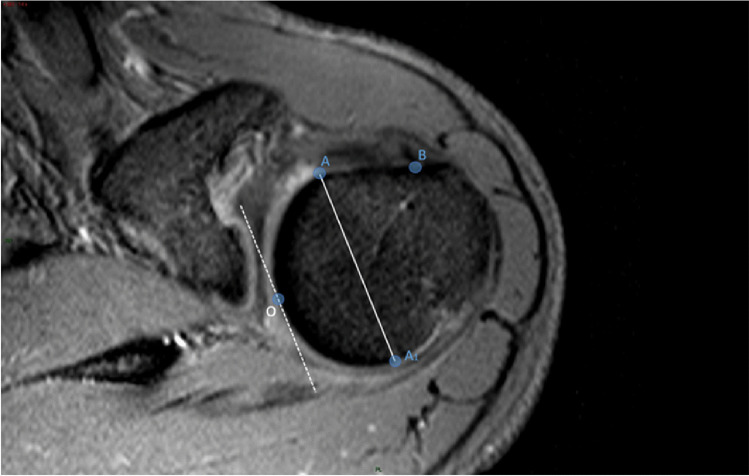
Axial T2w-FFE sequence showing the straight line drawn to connect the landmark A with the landmark A1, and the second straight line drawn (dashed in the figure), with a course parallel to the first line and tangent to the sphericity of the humeral head. The tangency point corresponds to the point of maximum convexity of the humeral head and, moreover, to the origin (O) of our reference system

**Fig. 4:**
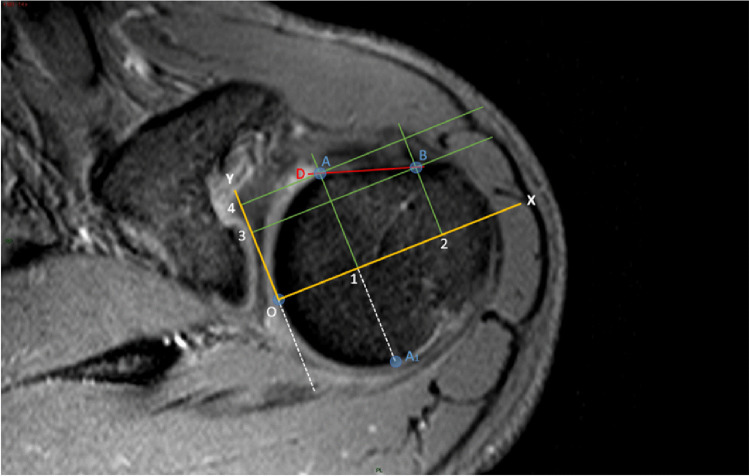
Axial T2w-FFE sequence showing the reference system used to identify the humeral head profile morphological variants. O represents the origin on the reference system; Cartesian XY (yellow lines) diagram is drawn with the X axis oriented along the major axis of the humeral head; 4 orthogonal axes (1–4 green lines) intersect the previously identified landmarks A and B; the diagonal D (red line) represents the horizon in the morphological analysis

Once the landmarks were identified, a straight line was drawn connecting the landmark A with the landmark A1 (Fig. 3). Then, a second straight line was drawn (dashed in the figure), with a course parallel to the first line and tangent to the sphericity of the humeral head. The tangency point corresponds to the point of maximum convexity of the humeral head and, moreover, to the origin (O) of our reference system (Fig. 4). After identifying the origin (O) on the reference system, a Cartesian XY diagram was drawn (yellow lines in Fig. 4) with the X axis oriented along the major axis of the humeral head (Fig. 4). Subsequently, 4 orthogonal axes were drawn (1–4 green lines in Fig. 4) that intersected the previously identified landmarks A and B. Finally, a diagonal D (red line in Fig. 4) was drawn which served as a horizon in the morphological analysis.

**Fig. 5:**
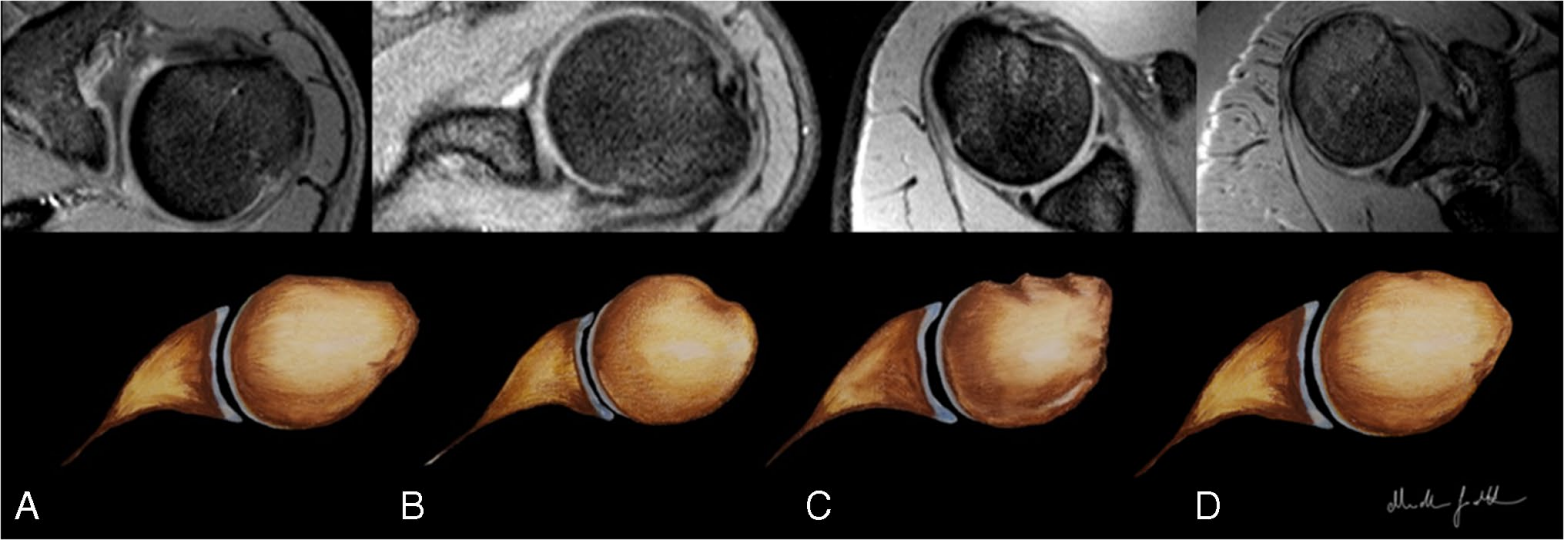
Four possible main morphological variants have been identified: 1. Flat shape; 2. convex shape; 3. spiculated shape; and 4. mixed morphology

Using this reference system, four possible main morphological variants were identified (Fig. 5): 1. flat shape; 2. convex shape; 3. spiculated shape; and 4. mixed morphology. A detailed description of the 4 morphological variants is shown in Table 3.

**Table 3:** Four variants of biceps pulley reflection area

Classification	Description
Flat shape	Bone profile parallel to the diagonal D
Convex shape	Bone profile curved anterior to the diagonal D
Spiculated shape	Bone profile that intersects the diagonal D in at least two points
Mixed shape	Bone profile with characteristics superimposable on both the convex and spiculated forms

### Statistical analysis

Categorical variables are described as frequencies with percentages. Intra-observer and inter-observer agreements were calculated using Cohen K test, with a 95% confidence interval. The values were interpreted according to the adapted guidelines of Landis and Koch. Excellent agreement occurred when the kappa value was between 0.81 and 1.00; good agreement between 0.61 and 0.80; moderate agreement between 0.41and 0.60; fair agreement between 0.21 and 0.40; and poor agreement less than 0.20 [5, 6]. All data were analyzed with GraphPad Prism (GraphPad Prism version 8.2.1 for macOS, GraphPad Software, San Diego, CA, USA).

## Results

Of the initial 326 MR shoulder examinations, 311 fulfilled the inclusion criteria. Fifteen patients were excluded from the statistical analysis because both examiners did not consider the assessment of the morphology of the biceps reflection pulley area reproducible due to poor diagnostic quality of the MRI exam or the presence of motion artifacts.

**Fig. 6:**
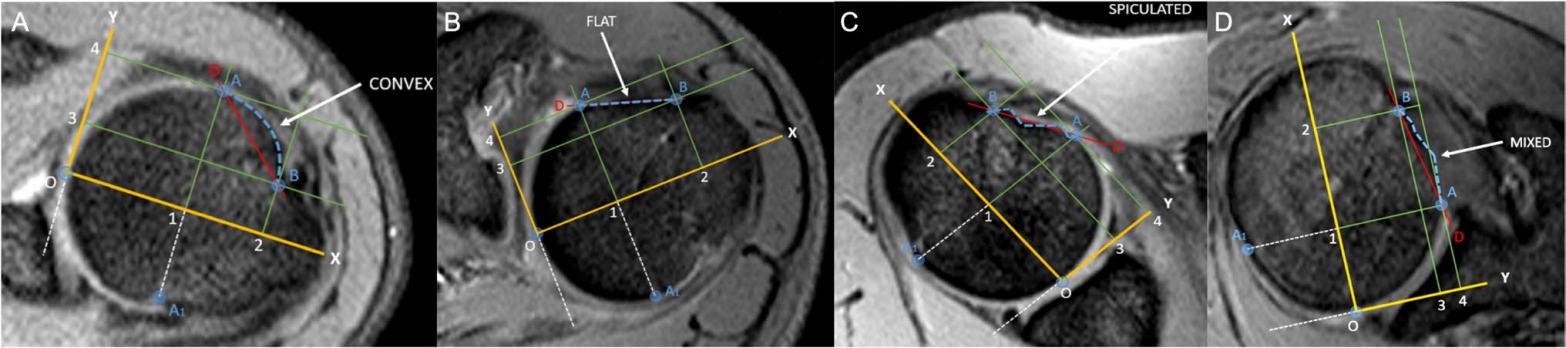
**A**, **B**, **C**, **D** Images obtained with axial T2w-FFE sequence show the convex shape in **A**, the flat shape in **B**, the spiculated shape in **C**, and the mixed morphology in **D**

The four main morphological variants were identified as follows: 95 cases (31%) with *convex* shape(Fig. 6A), 127 cases (41%) with *flat* shape (Fig. 6B), 77 cases (25%) with *spiculated* shape (Fig. 6C), and 12 cases (4%) with *mixed* morphology (Fig. 6D).

**Fig. 7:**
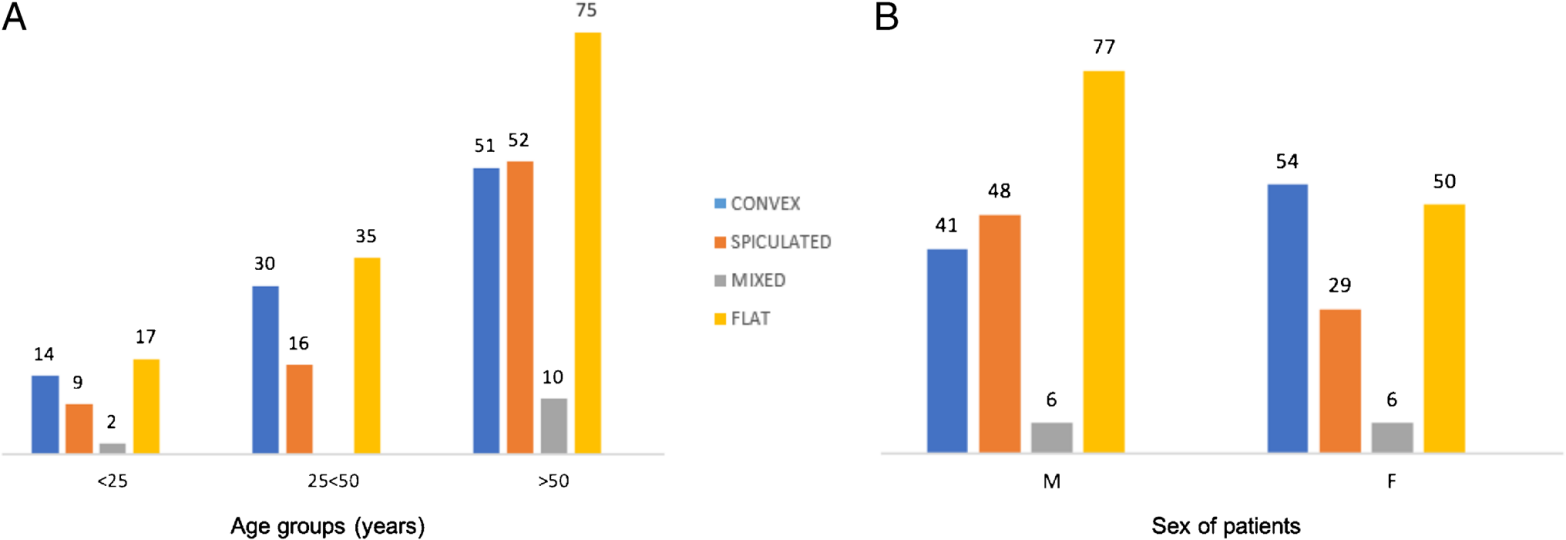
**A** The histogram shows the distribution of the morphological variants based on age groups. **B** The histogram shows the morphological variants distribution based on gender

Figure 7A shows the distribution of the morphological variants based on age groups. In the age group under 25 years (42 shoulder examinations), 14 cases (33%) were classified as *convex* shape, 17 cases (41%) as *flat* shape, 9 cases (21%) as *spiculated* shape, and 2 cases (5%) as *mixed* morphology. In the age group between 25 and 50 years (81 shoulder examinations), 30 cases (37%) a bicipital reflection pulley area with *convex* shape, 35 cases (43%) with *flat* shape, and 16 cases (20%) with *spiculated* shape. In this group, no patients had a *mixed* morphology. In the age group over 50 years (188 shoulder examinations), 51 cases (47%) were classified as *convex* shape, 75 cases (40%) as *flat* shape, 52 cases (28%) as *spiculated* shape, and 10 cases (5%) as *mixed* morphology.

Figure 7B shows the morphological variant distribution based on gender. In the male group (172 shoulder exams), 41 cases (24%) with *convex* shape were identified, 77 cases (45%) with *flat* shape, 48 cases (28%) with *spiculated* shape, and 6 cases (3%) with *mixed* morphology. In the female group (139 shoulder exams), 54 cases (39%) with *convex* shape were identified, 50 cases (36%) with *flat* shape, 29 cases (21%) with *spiculated* shape, and 6 cases (4%) with *mixed* morphology. There was no statistically significant difference between shoulder morphological variants and demographic age and sex groups (*p* > 0.05).

The statistical analysis showed an excellent inter-observer agreement between radiologists (*k* = 0.887; 95% *CI*, 0.713–0.911) and also an excellent reproducibility for both observer 1 (*k* = 0.968; 95% *CI* 0.959–0.978) and observer 2 (*k* = 0.932, 95% *CI* 0.915–0.949).

## Discussion

The most commonly observed morphologic variant was a flat shape of the humeral head at the biceps pulley reflection. This result was found in the male group, in the age groups under 25 years and between 25 and 50 years. Both in the female group and in the age group over 50 years, we have found a slightly higher prevalence of the convex shape compared to the flat as well as in both these groups, we observed a relative greater frequency of spiculated shape. The biceps reflection pulley area with mixed morphology was by far the least frequently observed variant in our study population and in all its subgroups. Furthermore, no perfect agreement was found in the detection of this morphology by the two observers, since it was identified in 14 cases by the first observer and in 10 by the second. A final agreement was reached between the two musculoskeletal radiologists by assigning this type of morphology to 12 cases.

To the best of our knowledge, there is no previous study that investigates the morphological variants of biceps pulley reflection area. As mentioned above, the main purpose of this study was to identify possible anatomical variants of the morphology of the antero-superior profile of the humeral head at the reflection point of the biceps pulley.

The general dilemma of the shoulder as a synovial ball and socket joint is to provide high mobility and, at the same time, to preserve adequate stability. This reflects the controversial pathological-anatomical role of the rotator interval (RI) as it acts as a dynamic stabilizer on the one hand but, at the same time, represents a structural weakness point of the capsule providing the outlet for the LHBT [6, 7]. The position of the biceps tendon within the RI and the biceps sulcus represents an important factor for the stability of the LHBT reflection pulley. Indeed, a significant contribution to the painful symptomatology of the shoulder is given by the involvement of LHBT, and of the RI structures, considered elements of stability. Consequently, pathological changes of these structures could influence stability and mobility of the gleno-humeral joint [6, 7]. In some experimental studies, it has been shown that the partial section of the RI causes an increase in the anterior, lower, and posterior translation of the humeral head, while its surgical closure limits external rotation, elevation, and extension of the arm [9–11].

The prevalence of biceps pulley injuries is 7%, representing a significant source of morbidity [12]. Both traumatic and non-traumatic causes may result in injury to the reflection of the biceps pulley. Traumatic injuries usually occur due to a fall on an outstretched arm in combination with a complete extra- or intra-rotation or a fall backwards on the hand or elbow [13]. Non-traumatic injuries generally occur due to repetitive chronic activities such as ball-throwing sports including baseball, tennis, or volleyball [14].

One cause of repeated microtrauma causing pulley injury is the so-called antero-superior conflict. Gerber and Sebesta [14] were the first to describe intra-articular impingement. The lower surface of the subscapularis tendon and the biceps pulley collide with the anterior glenoid edge when the arm is in the position of horizontal adduction and internal rotation. Also, injuries of the rotator cuff, especially in the presence of very anterior insertion of the supraspinatus tendon and of the subscapularis, can be associated with lesions of the pulley with the involvement of CHL and SGHL [15]. Injuries in this region can cause biceps tendon instability resulting in subluxation or, in worst cases, dislocation of the biceps tendon [14, 16].

The term “hidden lesions” was described by Walch et al. [4], referring to tears of the subscapularis tendon, in presence of an intact biceps pulley or rotator interval, whose visualization during open surgery is difficult until the opening of the RI. Originally, this term was related to the difficulty in diagnosing pulley lesions by clinical tests, imaging, and even arthroscopy. Subluxation of the LHBT was defined by Walch et al. as a tendon dislocation above the medial edge of the intertubercular groove in its superior part. In their study, in case of subluxation, they found a torn SSC tendon with an intact pulley [13]. Walch et al. defined the dislocation of the LHBT as a complete and non-reducible loss of contact with the intertubercular groove. They classified the dislocations into four types: LHBT dislocation within the subscapularis tendon with an intact anterior fibrous fascia; intra-articular dislocation of biceps with complete tearing of all anterior muscle and ligaments but an intact anterior fascia; intra-articular dislocation of biceps with complete laceration of anterior fascia and of all insertions on lesser tuberosity; and dislocation over an intact SSC tendon with ruptures of CHL and SSP tendon.

Degenerative lesions of the rotator interval may be due to anatomical changes that cause LHBT instability [17–19]. LBHT instability can affect the intracapsular portion only, characterized by greater mobility of the tendon without loss of contact with the bone surface, but also its extracapsular component [20].

A prompt diagnosis of pulley lesions is required to identify intracapsular LHBT instability before rotator cuff tear occurs, and extracapsular instability to prevent subluxation and dislocation of LHBT.

In 2004, Habermeyer et al. [21] identified and described four different pulley injury groups and established a new classification system based on the importance of the SGHL at the lateral rotator interval. Group I includes isolated tear of the SGHL. Group II includes tears of the SGHL and tears of the adjacent SSP tendon. Group III includes tears of the SGHL and tears of the adjacent SSC tendon. Group IV includes tears of the SGHL and tears of the adjacent SSC and SSP tendons.

The instability of the LHBT can also depend on the length of the medial/lateral walls of the bicipital groove, on the opening/angles of the medial wall, on the width/depth of the bicipital groove, and on the presence of the supratubercular ridge [22–24]. During multidirectional biomechanical movements, longer walls should provide greater tendon stability in the bicipital groove than shorter ones. As the length of the medial and lateral walls decreases, instability increases and the tendon risks being inflamed and injured.

Several limitations of our study have to be considered. First, the study design is retrospective. Second, the sample size in our study is relatively small. Third, both groups of patients, with non-contrast MRI and MR arthrography examinations, were included in the study which might lead to spectrum bias. Fourth, it was not possible to obtain arthroscopic feedback of our results.

Our morphological analysis opens up to a series of questions that deserve further study. In fact, in this sense, the current work was planned to explore basic data concerning morphological variants of biceps pulley reflection; this will be followed by a further study focused on verifying whether these morphological variants may be potential predisposing factors for the micro-instability of the LHBT which could therefore play an essential role in the understanding of the kinematics of the tendon related to the lesions of the pulley. Furthermore, to our knowledge, it is unknown whether the chondral print, an indirect arthroscopic sign of LHBT instability identified by Castagna et al. [25] and characterized by cartilage erosion caused from the hypermobility of the tendon in its intra-articular portion, correlates with the results of our study. This study could represent a starting point from which to add further elements in understanding the instability of the LBHT.

In conclusion, MR of the shoulder provides high accuracy in the evaluation of the morphological variants of biceps pulley reflection area and we recommend that this analysis should be preferably assessed on axial images.
